# Concurrent chemo-radiotherapy with S-1 as an alternative therapy for elderly Chinese patients with non-metastatic esophageal squamous cancer: evidence based on a systematic review and meta-analysis

**DOI:** 10.18632/oncotarget.16302

**Published:** 2017-03-16

**Authors:** Guo-Min Song, Xu Tian, Xiao-Ling Liu, Hui Chen, Jian-Guo Zhou, Wei Bian, Wei-Qing Chen

**Affiliations:** ^1^ Department of Nursing, Tianjin Hospital, Tianjin, China; ^2^ Department of Gastroenterology, Chongqing Cancer Institute & Hospital & Cancer Center, Chongqing, China; ^3^ Department of Oncology, Affiliated Hospital to Zunyi Medical University, Zunyi, China; ^4^ Ophthalmology Department, Southwest Hospital, Third Military Medical University, Chongqing, China

**Keywords:** esophageal squamous cancer, gimeraciland oteracil porassium, chemo-radiotherapy, meta-analysis, GRADE

## Abstract

**Objective:**

This systematic review and meta-analysis aims to systematically assess the effects of concurrent chemo-radiotherapy (CRT) compared with radiotherapy (RT) alone for elderly Chinese patients with non-metastatic esophageal squamous cancer.

**Methods:**

We searched PubMed, EMBASE, Cochrane Central Register of Controlled Trials (CENTRAL), China Biomedical Literature Database (CBM), and China National Knowledge Infrastructure (CNKI) databases. We retrieved randomized controlled trials on concurrent CRT with Gimeraciland Oteracil Porassium (S-1) compared with RT alone for aged Chinese patients with non-metastatic esophageal squamous cancer performed until August 2016.

**Results:**

Eight eligible studies involving 536 patients were subjected to meta-analysis. As a response rate measure, a relative risk (*RR*) of 1.37 [95% confidence intervals (CIs): 1.24, 1.53; *P* = 0.00], which reached statistical significance, was estimated when concurrent CRT with S-1 was performed compared with RT alone. Sensitivity analysis on response rate confirmed the robustness of the pooled result. The *RR* values of 1.44 (95% CIs: 1.22, 1.70; *P* = 0.00) and 1.77 (95% CIs: 1.26, 2.48; *P* = 0.00) estimated for 1- and 2-year survival rate indices, respectively, were also statistically significant. The incidence of adverse events was similar in both groups.

**Conclusion:**

This review concluded that concurrent CRT with S-1 can improve the efficacy and prolong the survival period of elderly Chinese patients with non-metastatic esophageal squamous cancer and does not significantly increase the acute adverse effects of RT alone.

## INTRODUCTION

Esophageal cancer is a malignant condition involving the primary esophageal epithelium. Approximately 90%-95% of all esophageal cancers are categorized into squamous cell cancer [1]. Esophageal squamous cancer has been listed as the eighth common malignant cancer and the sixth contributor to cancer deaths [2]. Approximately 400,000 new cases of esophageal cancers are diagnosed annually worldwide [3]. The number of elderly patients with esophageal squamous cancer has greatly increased due to increased life expectancy and aggravated aging process [4, 5]. Studies have illustrated that the proportion of elderly patients is approximately 20% of all patients with esophageal cancer [6].

Age is a key factor considered in selecting appropriate treatment regime for esophageal squamous cancer, whose prognosis has been improved with development in surgery and radiotherapy (RT) approaches [7]. As the standard treatment regime for elderly patients with esophageal squamous cancer has not been established [5, 8], only 10%-15% of elderly patients with esophageal squamous cancer can survive 5 years after received RT [7–9]. In China, the treatment options for these given population were mainly designed based on the recommendations listed in National Comprehensive Cancer Network (NCCN) guideline [5, 9].

Gimeraciland Oteracil Porassium (S-1) is a novel anti-cancer agent derived from 5-FU and consists of tegafur, gimeracil, and oteracil potassium [10]. Evidence has suggested that S-1 exhibits higher anti-tumor activity, lower side effects, and excellent biological availability compared with other techniques based on Fu [11–14]. Published studies have suggested that concurrence of chemoradiotherapy (CRT) with S-1 effectively improved the clinical outcomes of elderly patients with esophageal squamous cancer [7, 15, 16]. Nevertheless, studies conducted only involved small sample sizes.

A systematic review and meta-analysis was performed to evaluate the potential of concurrent CRT with S-1 compared with RT alone for aged Chinese patients with non-metastatic esophageal squamous cancer. Subgroup analysis of survival time was also conducted. The quality of the evidence was comprehensively assessed using the Grading of Recommendations Assessment, Development, and Evaluation (GRADE) profiler to facilitate clinical decision making.

## RESULTS

### Study selection

**Figure 1:**
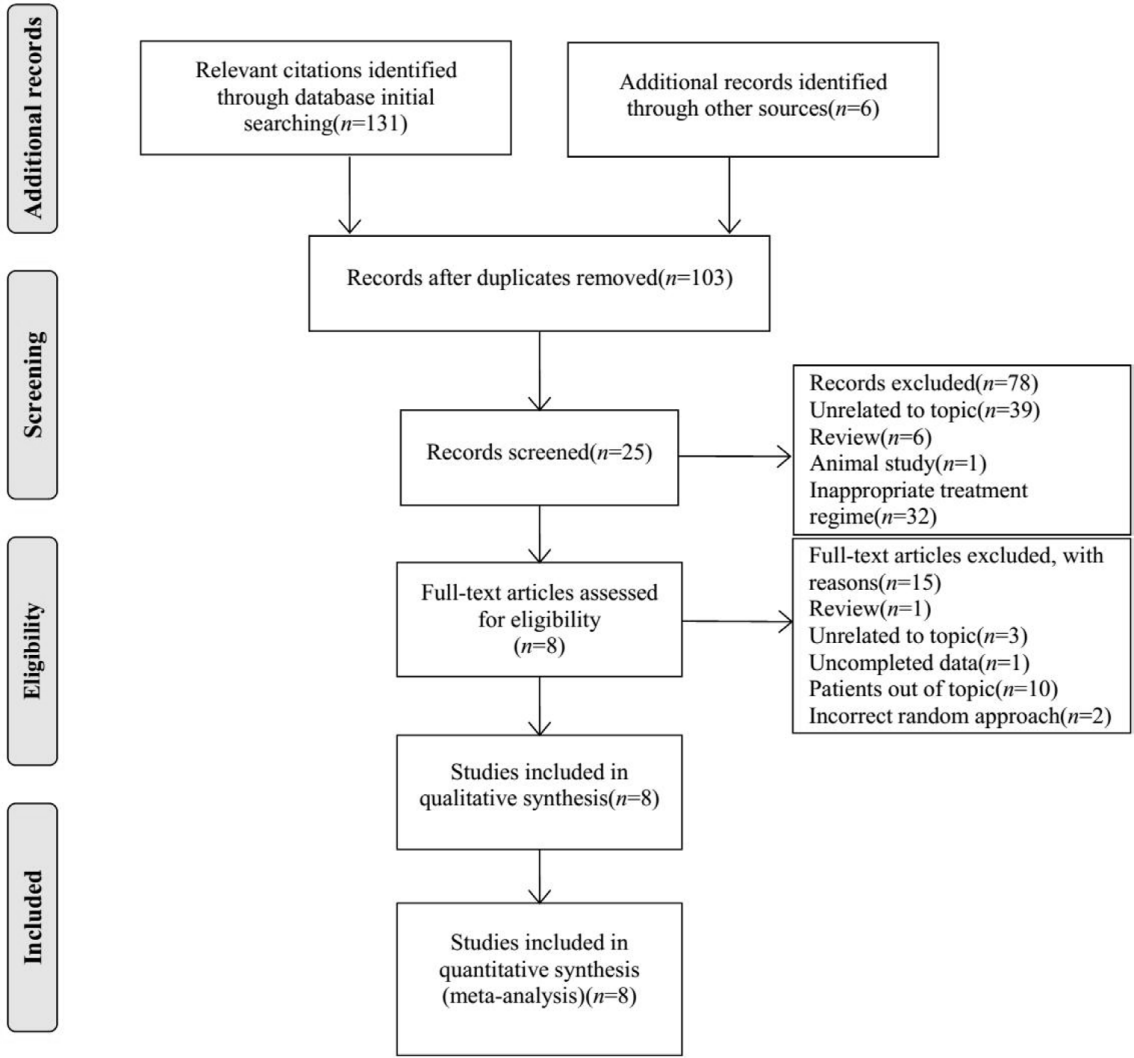
Flow diagram of citation retrieval and screening

We initially identified 131 potential citations through electronic searching of target databases, and six additional citations were included through other sources. Seventy-eight ineligible studies were excluded after screening study design, title, and abstract because of the following reasons: un-relatedness to the topic, review, animal experiment, and inappropriate treatment regime. Eight studies [11, 17–23] involving 536 patients were incorporated into the systematic review and meta-analysis, and the full text of these studies were retrieved. The flow diagram of citation retrieval and selection is presented in Figure 1.

### Basic characteristics of eligible studies

We designed a table to summarize the essential information and basic characteristics of all eligible studies (Table 1). All trials included were published between 2012 and 2014 and conducted in mainland China. Trials performed in Western countries were not eligible for this study. The sample size of all eligible studies ranged from 49 to 80. Seven studies described in detail the total dose of RT used [11, 18–23], which ranged from 50.4 Gy to 70 Gy. S-1 combined with 3D conformal radiotherapy, S-1 combined with intensity modulated radiation therapy, and S-1 combined with image-guided intensity-modulated radiotherapy were adopted in six [17–21, 23], one [23], and one [23] studies, respectively. All the studies reported response rate measures [11, 17–23], five studies used 1-year survival rate [17, 18, 20, 21, 23], and four studies reported 2-year survival rate [17, 20, 21, 23]. Data of adverse events were reported in seven studies [11, 17–19, 21–23].

**Table 1:** Basic characteristics of 8 studies included into this systematic review and meta-analysis

Study ID	Country	Diagnosis	Participants age (T/C)(years)	Number of patients (T/C)	Interventions in Treatment group	Interventions in Control group	Reported outcomes
Jiang XD 2012^16^	China	Esophageal squamous cancer	(65-78)/(67-77)	40/40	Intensity modulated radiotherapy (IMRT) with total dose of 60 Gy/30 f/6 months combined with S-1 with dose of 60 mg·m^-2^·d^-1^. S-1 was administered continued for 3 sessions and each session is consisting of medication-taking duration of 28 days and rest duration of 14 days.	Radiotherapy with total dose of 60 Gy/3 f/6 months.	Response Rate, Adverse Events
Lv SL 2014^17^	China	Esophageal squamous cancer	(70-76)/(71-77)	25/25	Intensity modulated radiotherapy (IMRT) combined with S-1 with 80 mg/(m^2^·d), twice a day, and orally on day 1-14 with 21 days as a cycle, two cycles as a whole.	Intensity modulated radiotherapy (IMRT) alone.	Response Rate, One-year survival rate, Two-year survival rate, Adverse Events
Song LQ 2014^18^	China	Advanced esophageal squamous cancer	≥65	40/40	Radiotherapy with 60-64 Gy/30-32 f combined with S-1 with 80 mg/(m^2^·d), twice a day, and orally on day 1-14 with 21 days as a cycle, two cycles as a whole.	Radiotherapy with 60-64 Gy/30-32 f alone.	Response Rate, One-year survival rate, Adverse Events
Song TT 2014^19^	China	Localized advanced esophageal squamous cancer	(60-74)/(61-75)	25/24	Radiotherapy with total dose of 60-66Gy combined with S-1 with 40 mg/(m^2^·d), twice a day, and orally on day 1-14 with 21 days as a cycle, two cycles as a whole.	Radiotherapy with total dose of 60-66Gy.	Response Rate, Adverse Events
Wang GM 2013^20^	China	Localized advanced esophageal squamous cancer	(60-78)/(63-81)	40/34	Radiotherapy with 60-70 Gy, fifth a week combined with S-1 with 40-60 mg/(m^2^·d), twice a day, and orally on day 1-14 with 21 days as a cycle, two cycles as a whole.	Radiotherapy with 60-70 Gy, fifth a week.	Response Rate, One-year survival rate, Two-year survival rate, Adverse Events
Wang XQ 2013^21^	China	Esophageal squamous cancer	(70-80)	30/30	Radiotherapy with 56-64 Gy/28-32 f combined with S-1 with 60 mg/(m^2^·d), twice a day, and orally on day 1-14 with 21 days as a cycle, two cycles as a whole.	Radiotherapy with 56-64 Gy/28-32 f alone.	Response Rate, One-year survival rate, Two-year survival rate, Adverse Events
Yang CL 2014^22^	China	Esophageal squamous cancer	(65-78)	31/32	Radiotherapy with 60 Gy/30 f/6 weeks combined with S-1 with 60 mg/(m^2^·d), twice a day, and orally on day 1-14 with 21 days as a cycle, two cycles as a whole.	Radiotherapy with 60 Gy/30 f/6 weeks.	Response Rate, Adverse Events
Zhang YX 2012^23^	China	Esophageal squamous cancer	≥60	40/40	Radiotherapy with 63 Gy/35 f combined with S-1 with 60 mg/(m^2^·d), twice a day, and orally on day 1-14 with 21 days as a cycle, two cycles as a whole.	Radiotherapy with 50.4 Gy/28 f.	Response Rate, One-year survival rate, Two-year survival rate, Adverse Events

Abbreviations: T: treatment group, C: control group

### Quality of methodology

**Figure 2:**
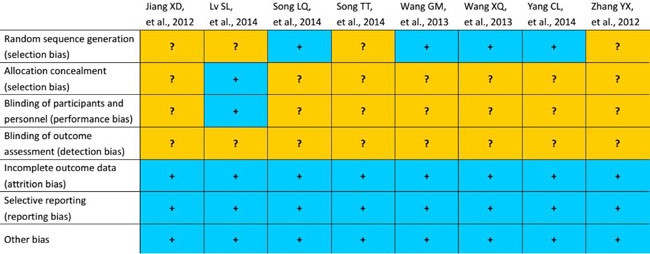
Assessment of risk of bias The yellow and blue represent “unclear risk of bias”, and “low risk of bias” respectively.

We evaluated the quality of the methodology of the studies included by assessing risk of bias. The Cochrane Risk of Bias Tool was used to assess the risk of bias [24]. The following domains were assessed by independent assessors: selection, performance, detection, attrition, reporting, and other biases [24]. The results of risk of bias assessment for each study are presented in Figure 2. Random sequence generation was performed in four studies [18, 20–22]. Only the study of Lv et al [17] performed allocation concealment and blinding of participants and personnel. All eligible studies described the reasons for incomplete outcome data [23]. The overall methodological quality of all studies included was generally good and fair.

### Grades of evidence

We presented the quality of the evidence of each clinical measure in Table 2. Three eligible clinical outcomes involving response rate and 1- and 2-year survival rates were assessed in this systematic review, and meta-analysis was performed using the GRADE system. Response rate was designated as critical measure, and 1- and 2-year survival rates were listed as important outcomes. The overall level of evidence was moderate for response rate and 1- and 2-year survival rates.

**Table 2:** The quality of the evidence of each clinical measure

No of studies	Design	Quality assessment					No of patients		Effect		Quality	Importance
		Risk of bias	Inconsistency	Indirectness	Imprecision	Other considerations	Radiotherapy combined with S-1	Radiotherapy alone	Relative(95% CI)	Absolute		
**Response Rate**												
8	randomised trials	no serious risk of bias	no serious inconsistency	no serious indirectness	no serious imprecision	reporting bias^1^	232/273 (85%)	166/269 (61.7%)	RR 1.37 (1.24 to 1.53)	228 more per 1000 (from 148 more to 327 more)	⊕⊕⊕O MODERATE	CRITICAL
>**One-year Survival Rate**												
5	randomised trials	no serious risk of bias	no serious inconsistency	no serious indirectness	no serious imprecision	reporting bias^1^	132/173 (76.3%)	87/165 (52.7%)	RR 1.44 (1.22 to 1.70)	232 more per 1000 (from 116 more to 369 more)	⊕⊕⊕O MODERATE	IMPORTANT
>**Two-year Survival Rate**												
3	randomised trials	no serious risk of bias	no serious inconsistency	no serious indirectness	no serious imprecision	reporting bias^1^	60/110 (54.5%)	31/101 (30.7%)	RR 1.77 (1.26 to 2.48)	236 more per 1000 (from 80 more to 454 more)	⊕⊕⊕O MODERATE	IMPORTANT

^1^Begg rank correlation test and Egger linear regression test generated the asymmetry of funnel plot

### Response rate

**Figure 3:**
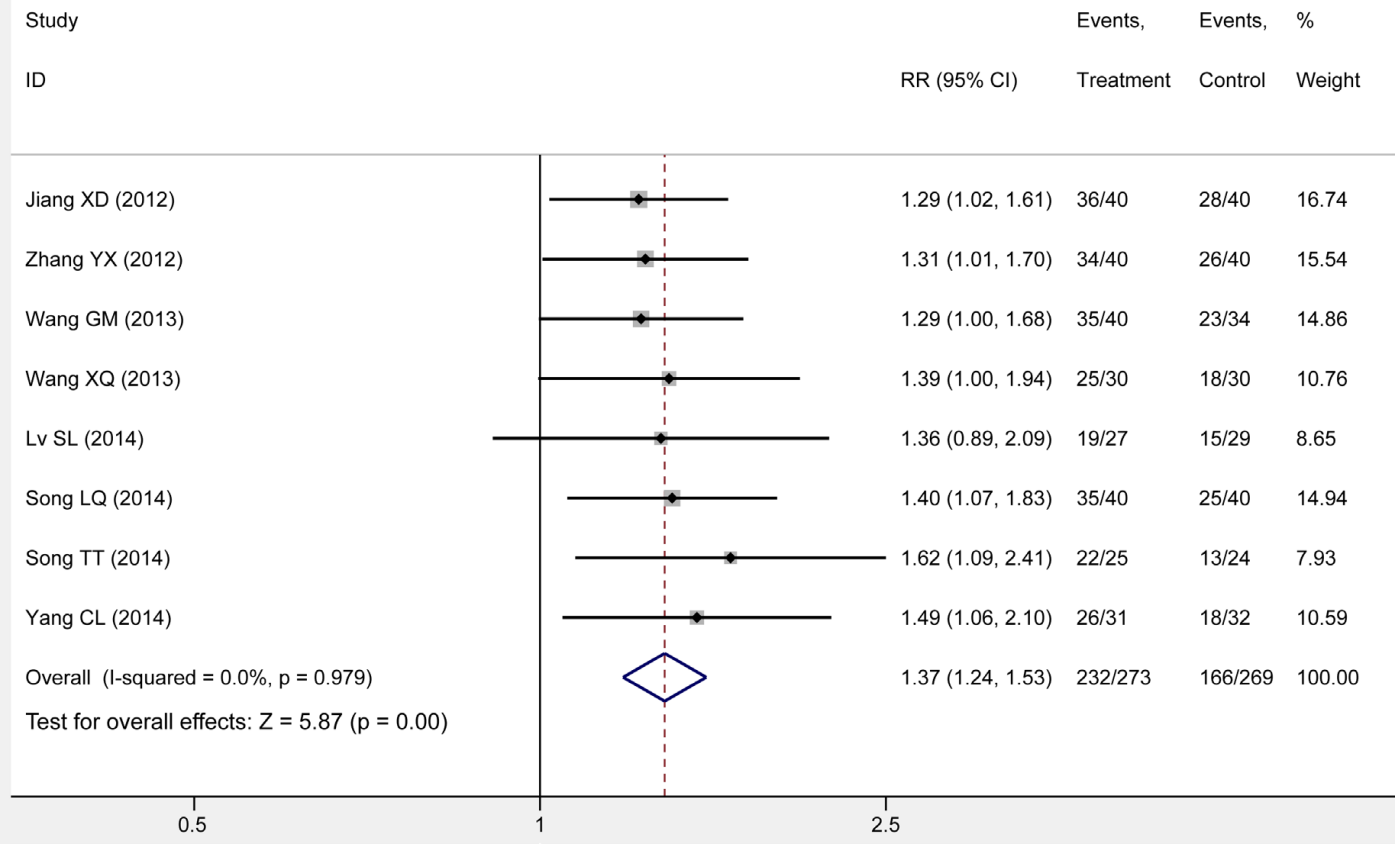
Meta-analysis on response rate The summary effect estimate (risk ratio, RR) for individual randomized controlled trials (RCTs) are indicated by grey rectangles (the size of the rectangle is proportional to the study weight), with the black horizontal lines representing 95% confidence intervals (CIs). The overall summary effect estimate (risk ratio) and 95% confidence interval are indicated by the blue diamond below.

All eligible studies [11, 17–23] reported response rate when S-1 combined with RT was performed relative to RT alone. The response rate of S-1 combined with the RT arm ranged from 81.25% to 90.00%, and that of the RT alone ranged from 54.20% to 70.00%. The pooled result suggested that the *RR* was 1.37 (95% *CI*: 1.24, 1.53; Z = 5.87, *P* = 0.00) with statistical significance (Figure 3) (*I*2 = 0.0, *P* = 0.979).

### Subgroup analysis on survival rate

**Figure 4:**
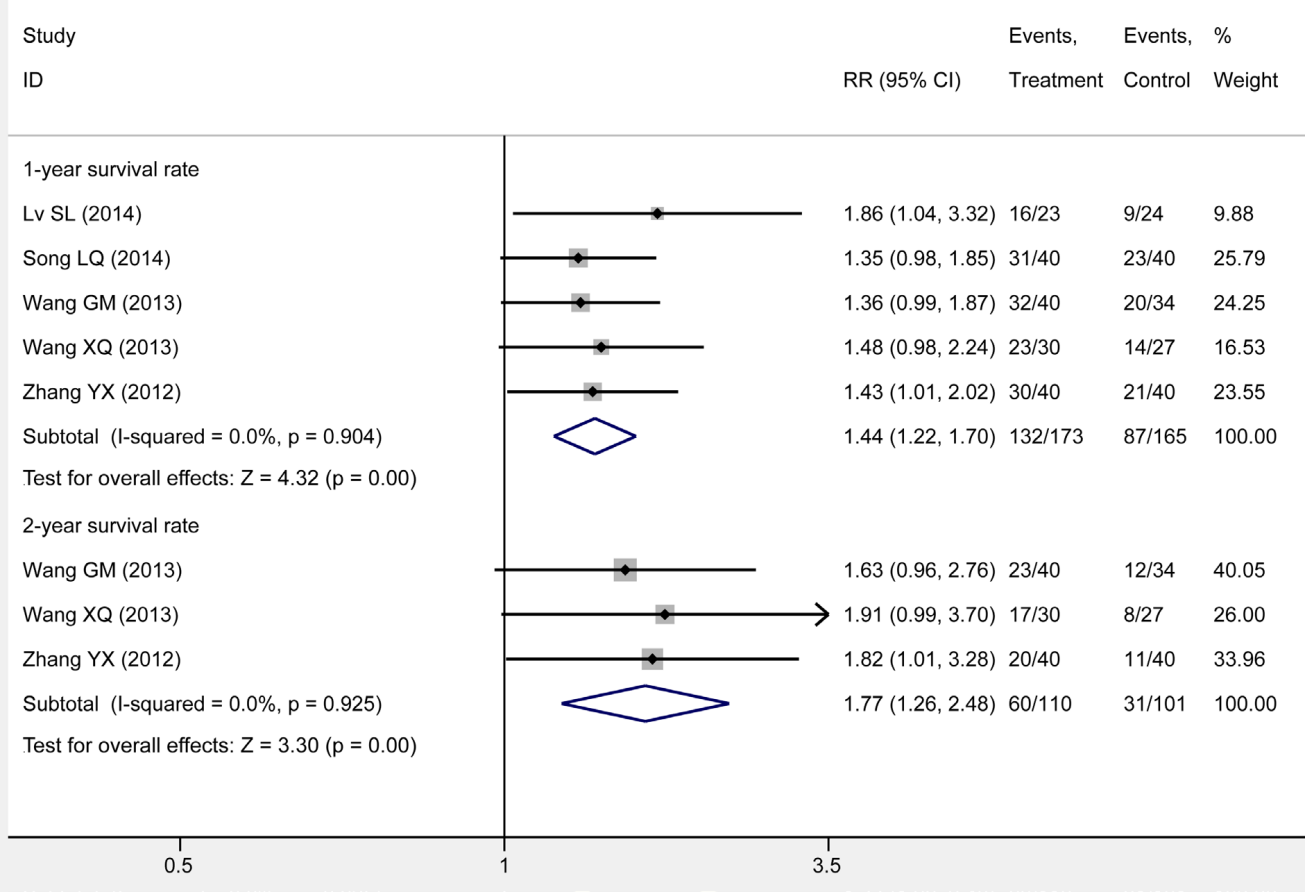
Meta-analysis on survival rate through subgroup analysis The summary effect estimate (risk ratio, RR) for individual randomized controlled trials (RCTs) are indicated by grey rectangles (the size of the rectangle is proportional to the study weight), with the black horizontal lines representing 95% confidence intervals (CIs). The overall summary effect estimate (risk ratio) and 95% confidence interval are indicated by the blue diamond below.

We performed a subgroup meta-analysis on the survival rate of various years. Five studies [17, 18, 20, 21, 23] reported the 1-year survival rate of the patients and were pooled to examine the effect of S-1 combined with RT compared with RT alone in elderly Chinese patients with non-metastatic esophageal squamous cancer. The results of meta-analysis showed that S-1 combined with RT effectively prolonged 1-year survival (*RR* 1.44; 95% *CI*: 1.22, 1.70; *Z* = 4.32, *P* = 0.00), with statistically significant difference from RT alone. No significant differences in clinical characteristic and methodology were identified for studies included, and no statistical heterogeneity was verified for all studies (*I*2 = 0.0, *P* = 0.904). Therefore, the fixed-effects model was used to pool effect size (Figure 4).

Four studies reported the 2-year survival rate of the patients [17, 20, 21, 23]. Differences in clinical characteristics and methodology were not apparent and no statistical heterogeneity was observed (*I*2 = 0.0, *P* = 0.925). Thus, the fixed-effects model was used to pool the data. The result indicated a significant difference among groups (*RR* 1.77; 95% *CI*: 1.26, 2.48; *Z* = 3.30, *P* = 0.00) (Figure 4).

### Adverse events

Seven eligible studies [11, 17, 19–23] reported the incidence of adverse events for both arms. We performed qualitative analysis to summarize the results because of the significant differences found in the studies included. Primary adverse events consisted of nausea, vomiting, radiation esophagitis, abnormal liver function, radiation pneumonitis. Two studies [21, 23] reported three-grade toxicities; however, the incidence of these adverse events without statistical significance between both arms (*P* > 0.05).

### Sensitivity analysis on response rate

**Figure 5:**
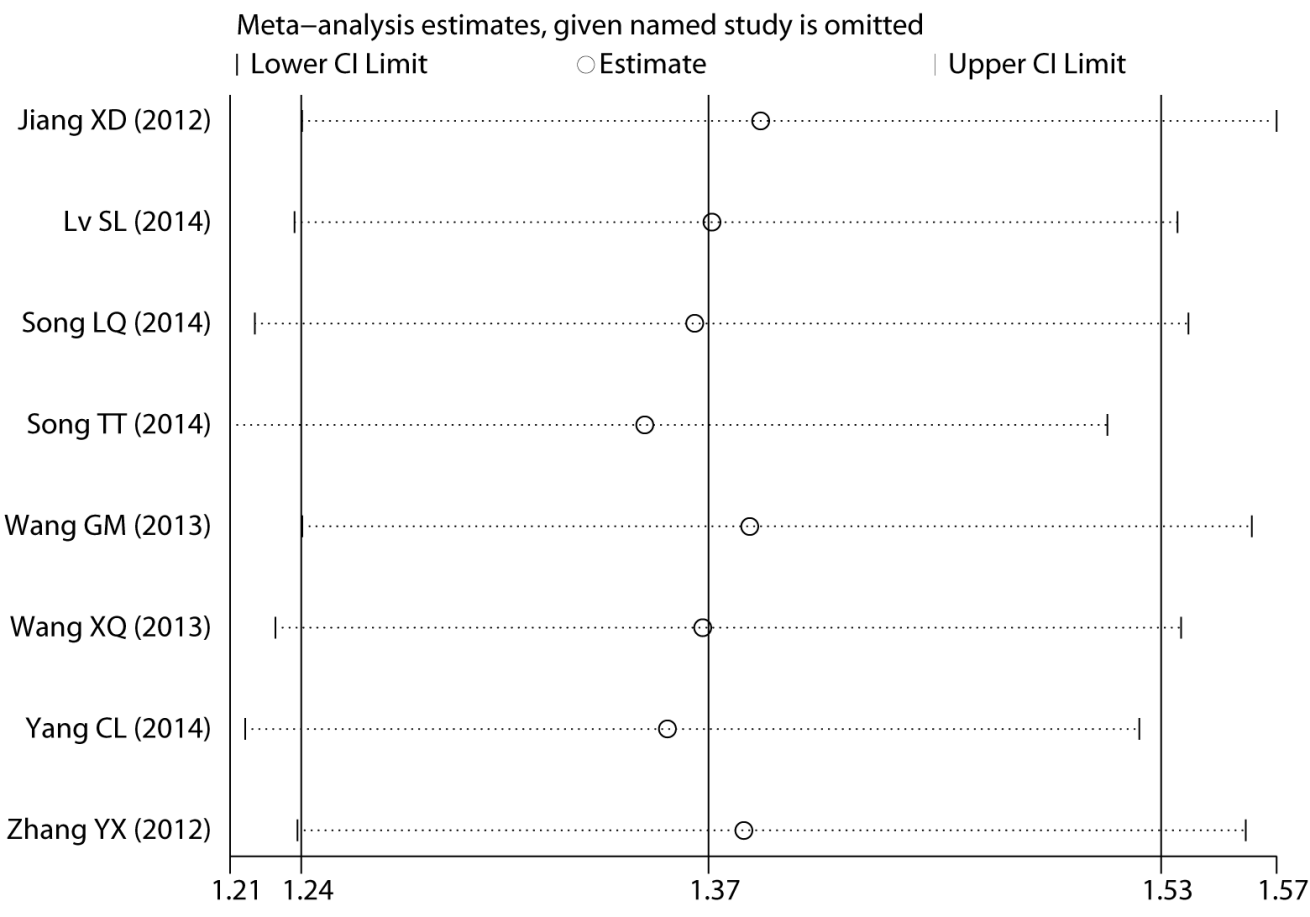
Sensitivity analysis on response rate

To determine the robustness of the pooled result on response rate, we performed a sensitivity analysis based on the leave-one-out approach [25, 26]. The result of sensitivity analysis confirmed the summarized result on response rate (Figure 5).

### Publication bias

**Figure 6:**
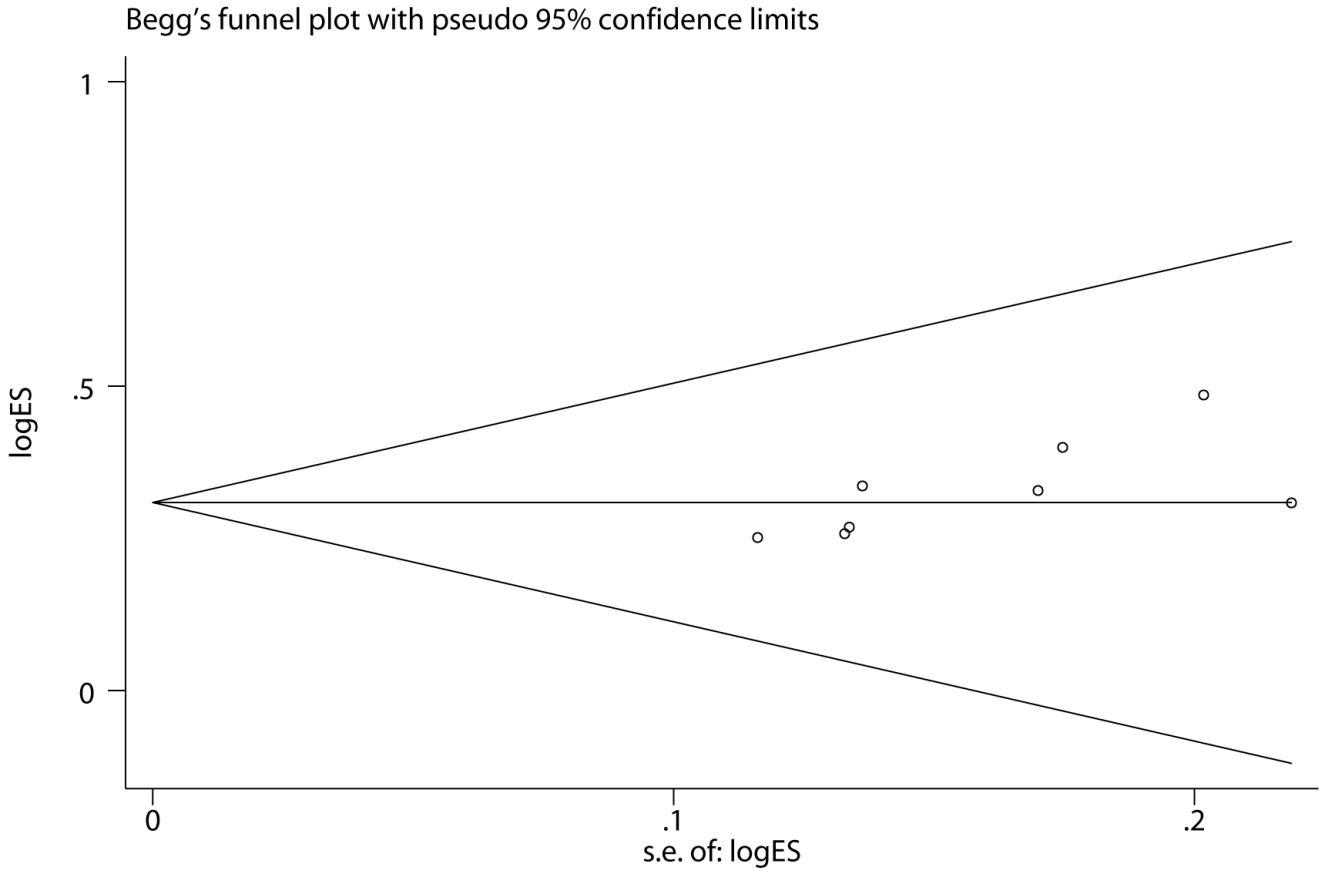
Begg's funnel plot for response rate The value presented in x axis indicated standard error of effect size and the value in y axis represented effect size.

**Figure 7:**
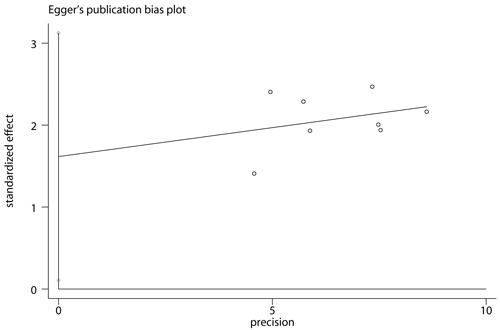
Egger's publication bias plot for response rate The value presented in x axis indicated precision and the value in y axis represented standardized effect.

We conducted Begg rank correlation and Egger linear regression tests to determine the publication bias of all eligible studies with the reported response rate [27]. The Begg's funnel plot (*z* = 2.10, *P* = 0.035) and Egger's publication bias (*t* = 2.62, *P* = 0.039) indicated that small sample size effect existed in these studies for measurement (Figures 6 and 7).

## DISCUSSION

Esophageal cancer is diagnosed in approximately half a million people annually, despite the development of various advanced medical techniques. The prognosis of this cancer is very poor [28]. Over the past decades, surgery was proverbially adopted to treat esophageal cancer because it can effectively improve the symptoms and prognosis of the patients [29]. However, application of surgery to elderly patients with esophageal cancer provided inadequate results because of numerous uncontrollable factors, such as multiple complications [5, 9]. Previous studies suggested that elderly patients with esophageal cancer have a mortality rate of 4.7%-7.2% after surgery [30–33]. Therefore, RT has been adopted to treat elderly patients with esophageal cancer but their 5-year survival remains lower after undergoing RT alone [9]. With the limitations of surgery and RT alone, concurrence CRT with 5-fluorouracil (5-Fu) (mainly refers to S-1) has been regarded as the optimal method to treat elderly patients with esophageal cancer [11, 15, 16, 34]. These options were also used in China because Chinese practitioners mainly designed treatment regime for elderly patients with esophageal cancer through considering recommendations proposed by NCCN guideline [5, 9, 11, 18, 19].

We performed a systematic review and meta-analysis to systematically evaluate the efficacy and safety of concurrence CRT with S-1 compared with RT alone for treatment of elderly Chinese patients with non-metastatic esophageal squamous cancer. The results of this study suggested that concurrence CRT with S-1 effectively increased the response rate relative to RT alone. For all individual eligible studies, although only four have generated significant findings, the direction of pooled result is consistent with the direction of single eligible study. Moreover, sensitivity analysis on response rate through excluding individual eligible study also conformed to the pooled result. And thus we are confident with the summarized result from the present meta-analysis.

For survival rate, we performed subgroup analysis based on different follow-up time. Our results indicated that RT with S-1 was associated with increased survival rate. In terms of 1-year survival rate, 2 of all 5 eligible studies generated significant findings. And we obtained more accurate result through accumulated sample size. For 2-year survival rate, 3 eligible studies were incorporated; however, none generated significant results. Nevertheless, spurious finding was avoided through accrued sample size, and a significant pooled result was generated in our meta-analysis, which was beneficial to concurrent CRT.

Based on the findings from our meta-analysis, we drawn a conclusion that concurrent CRT is an effective and safe alternative in increasing response rate and prolonging survival rate. Our conclusion is consistent with the recommendation proposed by NCCN guideline, which recommended concurrent CRT to be as the optimal alternative for treatment of esophageal cancer [8].

Although more accurate results were generated in our meta-analysis through accumulating sample size, several potential limitations existed in the present study were warranted to be acknowledged. First and perhaps the most important, only a small number of studies conducted in mainland China were included in this study, and Begg and Egger tests identified publication bias in these studies; and thus, reducing the power of the analyses. Second, although five main electronic databases, including PubMed, EMBASE, CENTRAL, CNKI, and CBM, were searched for potential citations, the exclusion of Science Direct, Springer Link, EBSCOhost, and Web of Knowledge may lead to risk of incompletely retrieved information in the present study. Thirdly, some unpublished and missing data may lead to bias to the pooled effect. Finally, because we only investigated the efficacy and safety of concurrent chemo-radiotherapy with S-1 for treatment of elderly Chinese patients with non-metastatic esophageal squamous cancer, and thus the findings from the present should be mainly translated into these given population. The researchers and practitioners must cautiously interpret the variance of different ethnic groups.

In conclusion, concurrent CRT with S-1 can improve the efficacy and prolong the survival of elderly Chinese patients with non-metastatic esophageal squamous cancer, without causing a significant increase in the acute adverse effects of RT alone. Consequently, concurrent CRT with S-1 can be an alternative therapy for elderly Chinese patients with non-metastatic esophageal squamous cancer.

## MATERIALS AND METHODS

This study was developed in accordance with the Preferred Reporting Items for Systematic Reviews and Meta-analysis statement [35] and Cochrane Handbook for Systematic Reviews of Interventions [36]. All statistical analyses were performed using data reported in previously published studies. Ethical approval and informed consents were not required.

### Identification of studies

Electronic databases including PubMed, EMBASE, Cochrane Central Register of Controlled Trials (CENTRAL), China Biomedical Literature Database (CBM), and Chinese National Knowledge Infrastructure (CNKI) were searched. We retrieved relevant randomized controlled trials (RCTs) on concurrent CRT with S-1 relative to RT alone for elderly Chinese patients with non-metastatic esophageal squamous cancer performed until August 2016. We used the following terms to search potential studies *via* target electronic databases: ‘Esophageal Neoplasms’, ‘Neoplasm*, Esophagea’, ‘Esophagus Neoplasm*’, ‘Neoplasms, Esophag*’, ‘Cancer of Esophagus’, ‘Cancer of the Esophagus’, ‘Esophagus Cancer’, ‘Cancer*, Esophagus’, ‘Esophageal Cancer*’; ‘S-1’; and ‘Randomized Controlled Trial’, ‘Randomized Controlled Trials as Topic’, ‘Controlled Clinical Trial’, ‘Controlled Clinical Trial as Topic’, and random*. The reference lists of eligible studies were also manually checked to identify other relevant trials. No language restriction was imposed to ensure recall ratio.

### Selection criteria

All potential citations were screened and cross-checked according to the following selection and exclusion criteria, which were developed based on the *PICOS* acronym: *a)* patients/participants (*P*): elderly Chinese patient was diagnosed as non-metastatic esophageal squamous cancer on the basis of histopathological examination, age of eligible patients greater than or equal to 70, and Karnofsky Performance Scale (KPS) scores greater than or equal to 70; *b)* intervention (*I*): concurrent CRT with S-1; *c)* comparison (*C*): radiotherapy alone; *d)* outcomes (*O*): response and survival rates and adverse events; and *e)* study design (*S*): RCTs.

Exclusion criteria included the following: *a)* patients with perforation of esophagus; *b)* patients suffering from distant metastasis; *c)* patients who cannot accept radiotherapy because of contraindication; *d)* animal and cell experiments; *e)* lack of essential information; *f)* repeated published studies or the same study but with different follow-time and research department, in which the article with the most strictest methodology and most complete data was incorporated into the article; *g)* non-original research, such as review, letter, and specialist comments.

### Data extraction

Two independent reviewers extracted the following information according to the pre-designed form: first author, publication year, country, sample size, patients’ baseline characteristics, diagnosis criteria, illness duration, study setting, and intervention. Continuous or binary data reported on specific outcomes were also obtained from original studies. The corresponding authors would be contacted to acquire relevant data when necessary. Any divergences in citation searching and screening, data extraction, assessment of the quality of methodology, data synthesis, and result interpretation were resolved by consulting a third author or achieving a consensus.

### Assessing risk of bias and grading the quality of evidence

Assessment of risk of bias of eligible studies was independently conducted by two reviewers by using the Cochrane Risk of Bias Tool [36]. Evaluation indices included randomization sequence generation, allocation concealment, blinding of participants, study personnel, and outcome assessors, incomplete outcome data, selective reporting, and other biases. Each domain was rated as “high risk,” “unclear risk,” or “low risk” based on the information extracted from primary studies.

The GRADE system was used to rate the levels of evidence with the following scoring protocol [24]: *a)* high: the estimate of effect approaches the true value and further research is unlikely to change the estimate; *b)* moderate: our confidence for the estimate of effect is rated as general and further research is likely to have an important effect on the estimate; *c)* low: inadequate confidence for the estimate of effect is identified and further research is very likely to change the estimate; and *d)* very low: any estimate of effect is very uncertain. The GRADE profiler software (version 3.6) (downloaded from: http://www.gradeworkinggroup.org/) was used to rate the level of evidence.

### Statistical analysis

All data extracted from eligible original studies were entered into the data editor for Windows in the STATA software (version 12.0; Stata Corp., College Station, TX) for statistical analysis. Clinical outcomes involving response rate, 1- and 2-year survival rates, and adverse events were evaluated. The first two parameters were synthesized quantitatively, whereas adverse events were qualitatively analyzed because of significant differences in the studies included. We subsequently calculated the relative risk (*RR*) and the corresponding 95% confidence interval (*CI*) because hazard ratios *(HR*) cannot be obtained from all eligible studies. Heterogeneity in the included studies was evaluated using Cochran's *Q* test, and the corresponding *P* value and substantial level of heterogeneity were evaluated using *I*2 statistic. Eligible studies with *I*2 ≥ 50% were considered heterogeneous, whereas studies with *I*2 *≤* 50% were considered homogeneous. Meta-analysis was performed on the clinical characteristics and methodology of eligible studies pooled by using the random-effects model or fixed-effects model based on the Mantel-Haenszel or inverse variance statistical approach. Subgroup analysis was also conducted on survival time. Sensitivity analysis was performed on response rate to investigate the robustness of the pooled result by using the leave-one-out approach. Publication bias of all studies included in the response rate measure was identified by performing Begg rank correlation and Egger linear regression tests.

## SUPPLEMENTARY MATERIALS FIGURE


