# Supplementary material for: Concurrent chemo-radiotherapy with S-1 as an alternative therapy for elderly Chinese patients with non-metastatic esophageal squamous cancer: evidence based on a systematic review and meta-analysis
Source: Oncotarget. 2017 Mar 16;8(23):37963–73. doi: 10.18632/oncotarget.16302 (PMC5514965; doi:10.18632/oncotarget.16302)
Supplement: Supplementary file 1 [file oncotarget-08-37963-s001.pdf]

Concurrent chemo-radiotherapy with S-1 as an alternative therapy for elderly Chinese patients with non-metastatic esophageal squamous cancer: evidence based on a systematic review and meta-analysis

Supplementary Material

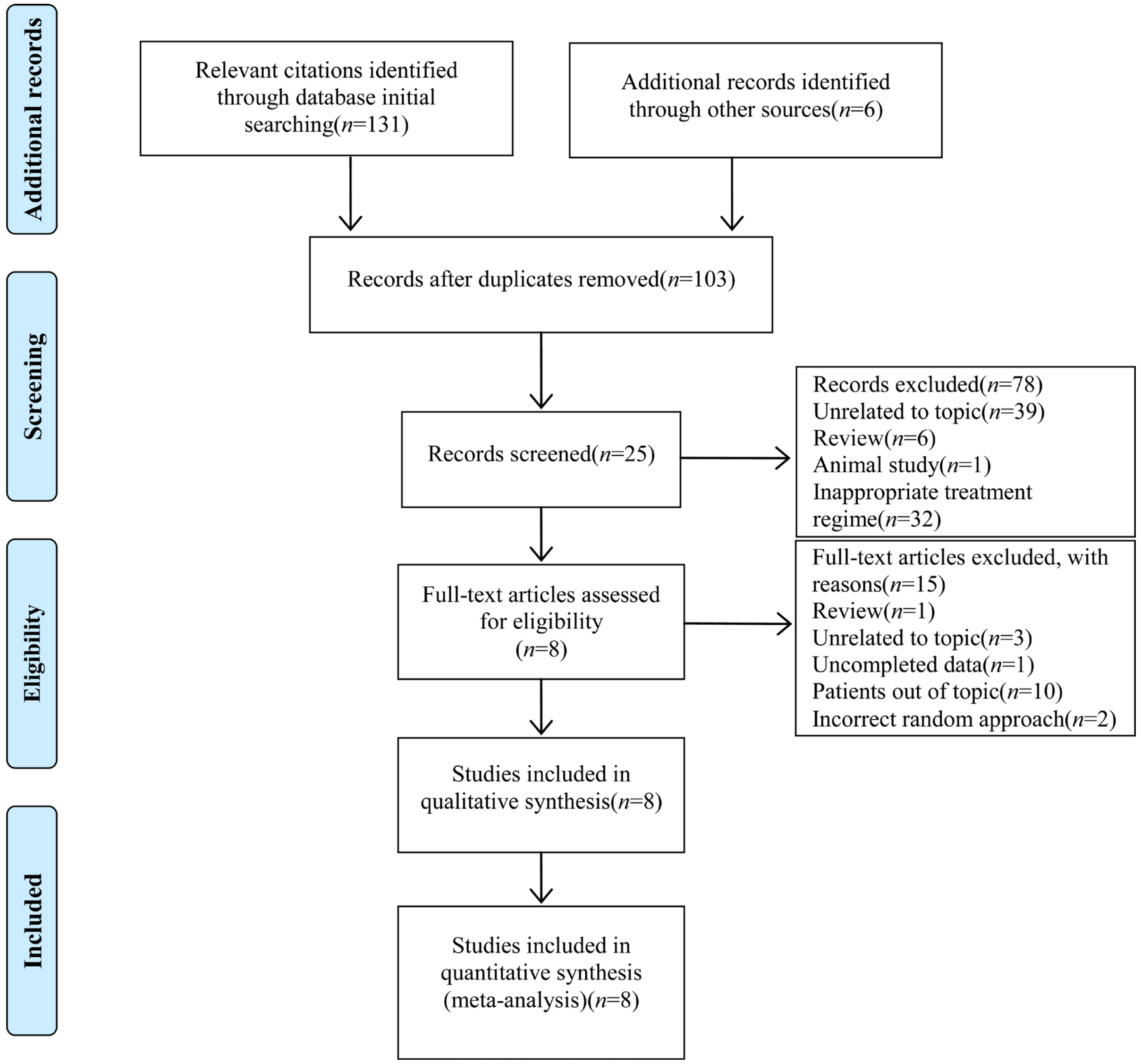

PRISMA 2009 Flow Diagram
